# Explainable machine learning framework to predict the risk of work-related neck and shoulder musculoskeletal disorders among healthcare professionals

**DOI:** 10.3389/fpubh.2024.1414209

**Published:** 2024-08-20

**Authors:** Na Luo, Xinyi Xu, Biling Jiang, Zeyuan Zhang, Jingyu Huang, Xiulan Zhang, Qiong Tan, Xuanyi Wang, Siyi Bai, Suyi Liu, Yishuang Pan, Chi Tang, Pinghua Zhu

**Affiliations:** ^1^Health Education and Promotion Department, Nanning Center for Disease Control and Prevention, Nanning, China; ^2^College of Humanities and Social Sciences, Guangxi Medical University, Nanning, China; ^3^Orthopedics Department, Nanning Hospital of Traditional Chinese Medicine, Nanning, China

**Keywords:** musculoskeletal disorders, machine learning, health care professionals, ergonomics, shiny app

## Abstract

**Objective:**

This study aims to develop risk prediction models for neck and shoulder musculoskeletal disorders among healthcare professionals.

**Methods:**

A stratified sampling method was employed to select employees from medical institutions in Nanning City, yielding 617 samples. The Boruta algorithm was used for feature selection, and various models, including Tree-Based Models, Single Hidden-Layer Neural Network Models (MLP), Elastic Net Models (ENet), and Support Vector Machines (SVM), were applied to predict the selected variables, utilizing SHAP algorithms for individual-level local explanations.

**Results:**

The SVM model excels in both Mean Absolute Error (MAE) and Root Mean Square Error (RMSE) and exhibits more stable performance when generalizing to unseen data. The Random Forest model exhibited relatively high overall performance on the training set. The MLP model emerges as the most consistent and accurate in predicting shoulder musculoskeletal disorders, while the SVM model shows strong fitting capabilities during the training phase, with occupational factors identified as the main contributors to WMSDs.

**Conclusion:**

This study successfully constructs work-related musculoskeletal disorder risk prediction models for healthcare professionals, enabling a quantitative analysis of the impact of occupational factors. This advancement is beneficial for future economical and convenient work-related musculoskeletal disorder screening in healthcare professions.

## Contributions to the literature

- Our study employs the Boruta algorithm for feature selection, reducing neck musculoskeletal disorder screening to just 12 key items and shoulder disorder screening to 17, enabling a simplified screening process. By inputting demographic data into an electronic system, the musculoskeletal disorder prediction model can assess the risk of these conditions in healthcare professionals, thereby significantly reducing the workload for screening.
- We have developed interpretable models for predicting the risk of shoulder and neck musculoskeletal disorders, utilizing SHAP algorithms for individual-level local explanations.
- Machine learning models show better prediction accuracy and precision compared to untrained Logistic regression, which was more commonly used in past research. Studies using machine learning for predicting musculoskeletal disorders in populations are relatively scarce.

## 1 Introduction

Work-related musculoskeletal disorders (WMSDs) refer to injuries or disorders of the muscles, nerves, tendons, joints, cartilage, and spinal disks that are associated with exposure to risk factors in the workplace (1). According to the data on work-related musculoskeletal disorders (WMSDs) from 2018 to 2020 published by the Chinese Center for Disease Control and Prevention, there are three high-prevalence groups in China: flight attendants, medical staff, and workers in vegetable greenhouses. Medical staff, in particular, are a high-risk group for WMSDs due to their heavy workloads accompanied by poor dynamic loads, static loads, physical loads, and ergonomic environments (2). Current research has revealed that WMSDs among medical staff are most commonly observed in the shoulder, neck, and lower back (3), with the highest prevalence occurring in the lower neck region (4).

Previous studies have predominantly utilized descriptive statistical analysis and logistic regression to analyze the influencing factors of musculoskeletal disorders among medical staff in terms of dynamic loading, static loading, physical loading, repetitive motion, ergonomic environment, and labor organization. Wang et al. employed logistic regression to analyze a sample of 1,017 medical staff in the department of obstetrics and gynecology and found that individual, postural, work-environmental, as well as psychosocial factors were the main contributors to musculoskeletal disorders (5). Krishnan et al. discovered that musculoskeletal disorders were associated with age, low education level, female gender, years of working experience, and lifestyle (6). Machine learning models have demonstrated significant advantages, such as high accuracy and resistance to overfitting. Consequently, they have been widely applied in predicting chronic diseases, infectious diseases, and tumors. However, the utilization of machine learning models in the study of work-related musculoskeletal disorders (WMSDs) remains relatively limited.

Considering these research gaps, we utilized data on Work-Related Musculoskeletal Disorders (WMSDs) from healthcare professionals in Nanning, Guangxi Zhuang Autonomous Region, to construct risk prediction models for shoulder and neck WMSDs. This approach quantitatively reveals the varying degrees of influence each variable has on the risk of developing work-related musculoskeletal disorders, A web calculator for the neck and shoulder disease risk of WMSDs was constructed based on shinyapps.io, which can be applied to the early detection and prevention of neck and shoulder WMSDs in healthcare workers. Risk prediction model for neck WMSDs website is: https://shoulderwmsdspred.shinyapps.io/neck/. Risk prediction model for shoulder WMSDs website of shoulder is: https://shoulderwmsdspred.shinyapps.io/shoulder/.

## 2 Methods

### 2.1 Setting and participants

This study, funded by the Health Commission of Nanning, was conducted as part of a survey on musculoskeletal disorders among occupational populations in Nanning. The research was carried out from June 2022 to March 2023. Medical personnel from medical institutions in seven districts and five counties of Nanning were selected as the study participants using stratified sampling. The survey was conducted online using the QuestionStar platform, and 617 medical personnel from three tertiary hospitals, seven secondary hospitals, and three disease control centers participated by completing the questionnaires.

### 2.2 Research tools

The questionnaire comprised four sections: personal information, musculoskeletal disorder status, work stress, and occupational health literacy. The Cronbach’s Alpha for this questionnaire is 0.741.

The musculoskeletal disorder status was assessed using Chinese version of the “Musculoskeletal Disorder Questionnaire” provided by the Occupational Health and Poison Control Institute of the Chinese Center for Disease Control and Prevention, a tool developed by referring to the musculoskeletal disorder survey forms in Nordic countries and adapted to the Chinese context (7). The survey assessed musculoskeletal disorders in nine areas: neck, shoulders, back, elbows, waist, wrists, hips, knees, and ankles/feet. The respondents reported neck and shoulder WMSDs occurrences during the last 12 months, which were used as dependent variables to construct neck and shoulder WMSDs predictive models.

The work stress scale utilized in this study was the Q17 Stress Test, which is widely applied to assess work stress in hospitals (8).

For evaluating occupational health literacy, the 2021 National Health Commission’s National Key Industry Occupational Health Literacy Monitoring Questionnaire was employed. A correct response rate of 60% was considered as having adequate occupational health literacy.

### 2.3 Ethical consideration

This study obtained approval from the Ethics Committee of Guangxi Medical University (approval number: 2021002). The purpose and content of the research were explained to all participants, and informed consent was obtained from each of them.

### 2.4 Machine learning model workflow

#### 2.4.1 Refining variables with the Boruta algorithm

The Boruta algorithm represents an approach for feature selection, particularly well-suited to address feature selection challenges within machine learning tasks. Its primary objective lies in the identification of the most pivotal attributes from a dataset teeming with numerous features, thereby bolstering model performance while mitigating the risk of overfitting.

**Figure 1:**
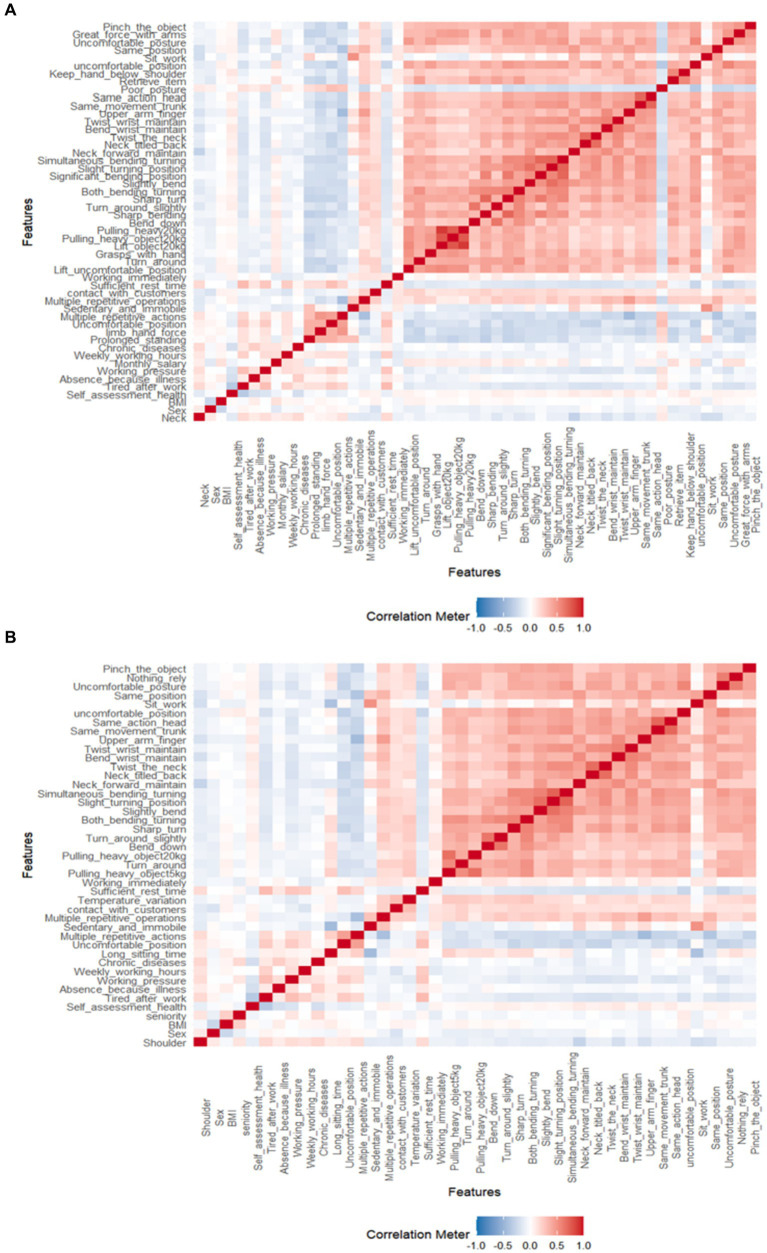
Heatmap illustrating the correlations between different variables. **(A,B)** Respectively present the variable correlations for musculoskeletal disorders in the neck and shoulder regions.

**Figure 2:**
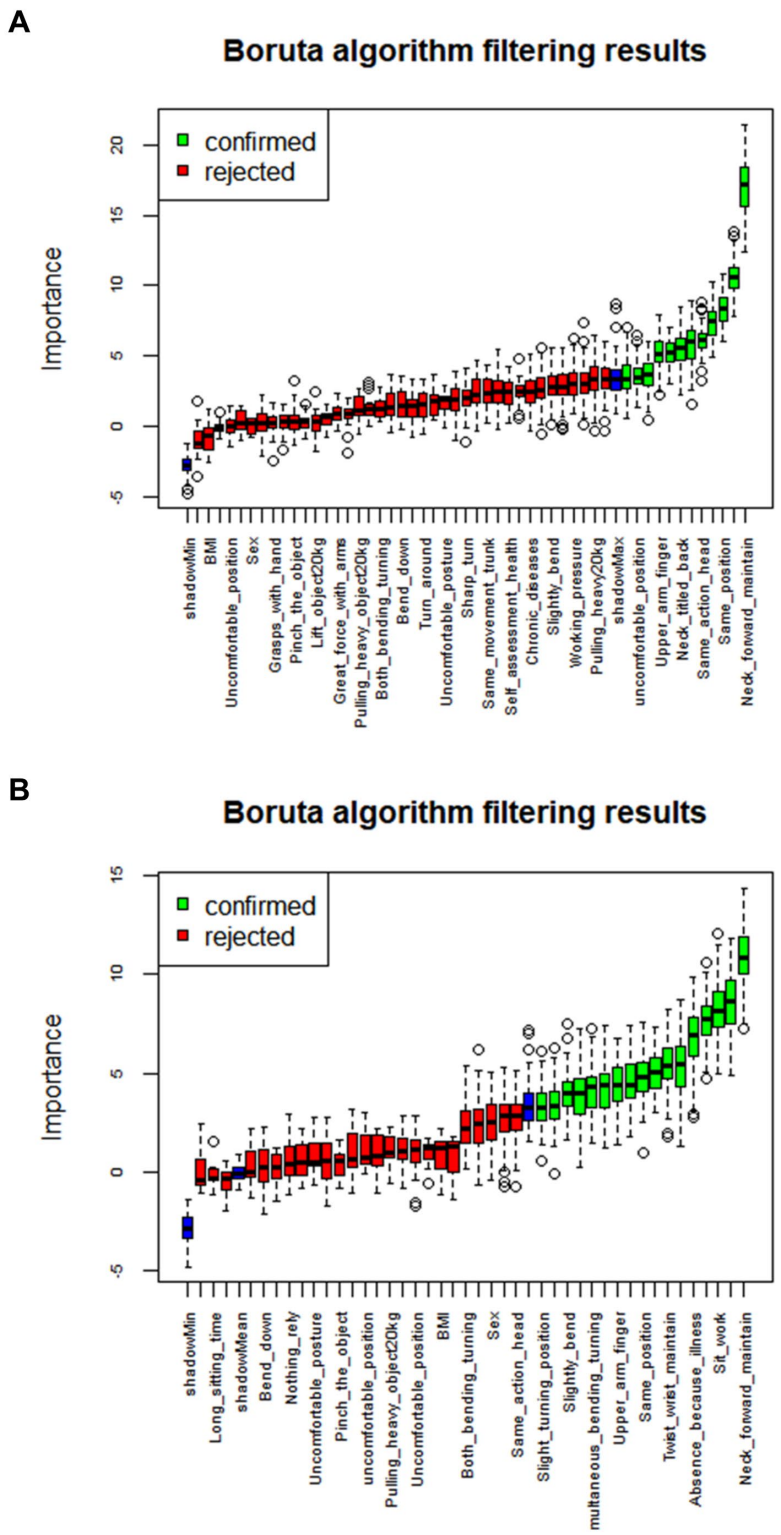
Data variable selection based on the Boruta algorithm. **(A,B)** Respectively depict the variable selection outcomes for the risk prediction dataset of neck musculoskeletal disorders and shoulder musculoskeletal disorders.

As indicated in Figure 1, it becomes evident that several variables exhibit pronounced interrelationships. In light of this observation, this research segregates the dataset into training and testing subsets at a 3:1 ratio. Subsequently, the target variables, namely the presence of neck and shoulder musculoskeletal disorders, are employed to train machine learning algorithms. Leveraging the Boruta algorithm, we undertake a rigorous examination of feature variables, culling those that bear no meaningful contribution to the model. Ultimately, this process yields 12 independent variables for the “Neck” category and 17 independent variables for the “Shoulder” category, as elaborated in Figure 2 and Table 1.

**Table 1:** Variables of the musculoskeletal disorder risk prediction model.

Abbreviation	Item	Variables of the work-related neck musculoskeletal disorder risk prediction model	Variables of the work-related shoulder musculoskeletal disorder risk prediction model
Tired_after_work	Do you feel physically tired after work?	√	√
Contact_with_customers	Does your job frequently involve interaction with patients or the public?	√	
Turn_around_slightly	Do you often slightly turn your body during work?	√	
Neck_forward_maintain	Do you frequently lean your neck forward or maintain this posture for extended periods during work?	√	√
Neck_titled_back	Do you frequently lean your neck backward or maintain this posture for extended periods during work?	√	√
Bend_wrist_maintain	Do you frequently bend your wrists or maintain this posture for extended periods during work?	√	√
Twist_wrist_maintain	Do you frequently twist your wrists and maintain this posture for extended periods during work?	√	√
Upper_arm_finger	Do you frequently repeat the same movements with your upper arms and fingers multiple times per minute during work?	√	√
Same_action_head	Do you frequently repeat the same movements with your head multiple times per minute during work?	√	
Uncomfortable_position	Do you often work in uncomfortable postures?	√	
Sit_work	Do you spend long periods sitting during work?	√	√
Same_position	Do you maintain the same posture for extended periods during work?	√	√
Self_assessment_health	Self-assessment of your health status		√
Absence_because_illness	Have you taken sick leave in the past year due to illness?		√
Working_pressure	Work-related stress		√
Chronic_diseases	Types of chronic diseases		√
Sufficient_rest_time	Do you feel your rest periods are sufficient?		√
Sharp_turn	Do you frequently make large turns of your body during work?		√
Slightly_bend	Do you frequently maintain a slightly bent posture for extended periods during work?		√
Slight_turning_position	Do you frequently maintain a slightly turned posture for extended periods during work?		√
Simultaneous_bending_turning	Do you frequently maintain a posture that involves both bending and turning for extended periods during work?		√

#### 2.4.2 Robustness assessment of models

**Figure 3:**
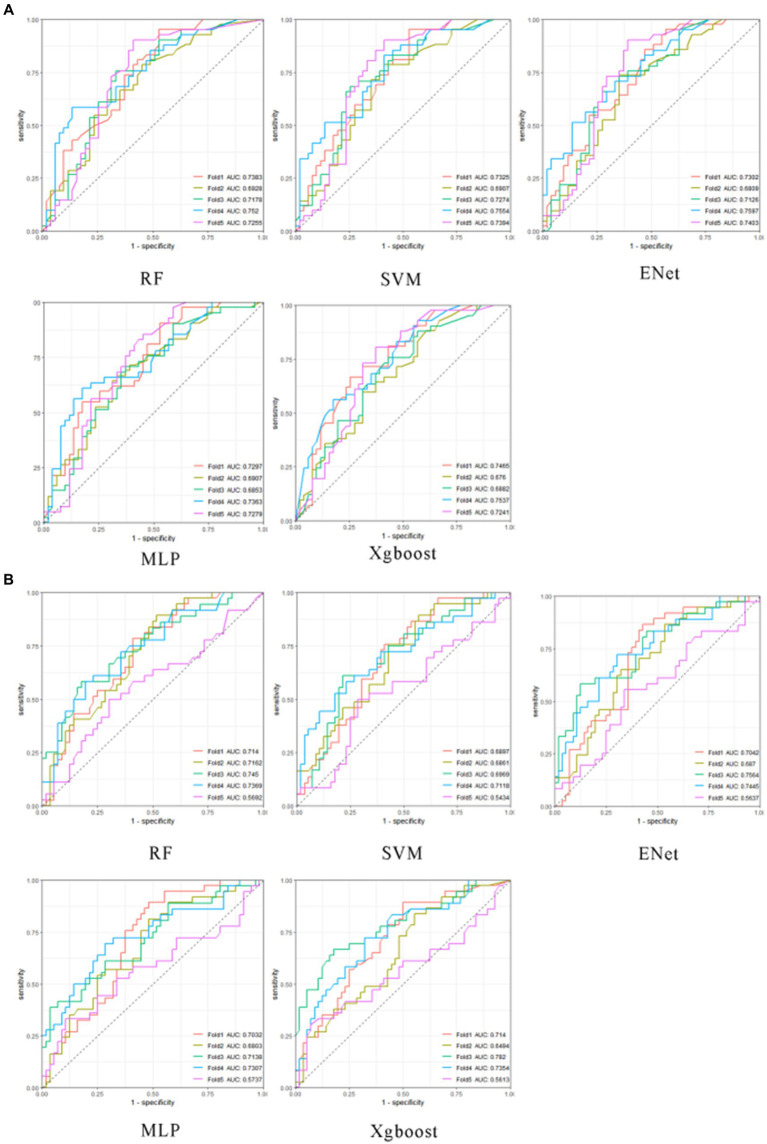
The optimal hyperparameter cross-validation results for machine learning models. The subplots in **(A)**, from left to right, and from the first row to the second row, represent the optimal hyperparameter cross-validation results for neck musculoskeletal disorder prediction models for RF, SVM, Enet, MLP, and Xgboost, respectively. The subplots in **(B)**, from left to right, and from the first row to the second row, represent the optimal hyperparameter cross-validation results for shoulder musculoskeletal disorder prediction models for RF, SVM, Enet, MLP, and Xgboost, respectively. The horizontal axis is sensitivity, and the vertical axis is 1-specificity.

We conducted a comparative analysis encompassing four distinct model categories: (1) Tree-Based Models: This category includes decision tree models, random forest models (RF), and XGBoost models (Xgboost). (2) Single Hidden-Layer Neural Network Models (MLP). The multilayer perceptron consists of multiple layers of neurons, where each layer is connected to the preceding layer, receiving its inputs. Simultaneously, each layer is also connected to the subsequent layer, influencing the neurons within the current layer. These layers include the input layer, hidden layer, and output layer. In this study, the MLP employed a single hidden layer comprising 15 hidden units. (3) Elastic Net Models (ENet). (4) Support Vector Machines (SVM). For each of these model categories, we performed an extensive hyperparameter grid search through 5-fold cross-validation on the training dataset (refer to Figure 3) (9). Subsequently, we evaluated model performance on both the training and testing datasets using metrics such as mean absolute error (MAE), root mean square error (RMSE), accuracy, and other relevant indicators.

#### 2.4.3 Model interpretability

**Figure 4:**
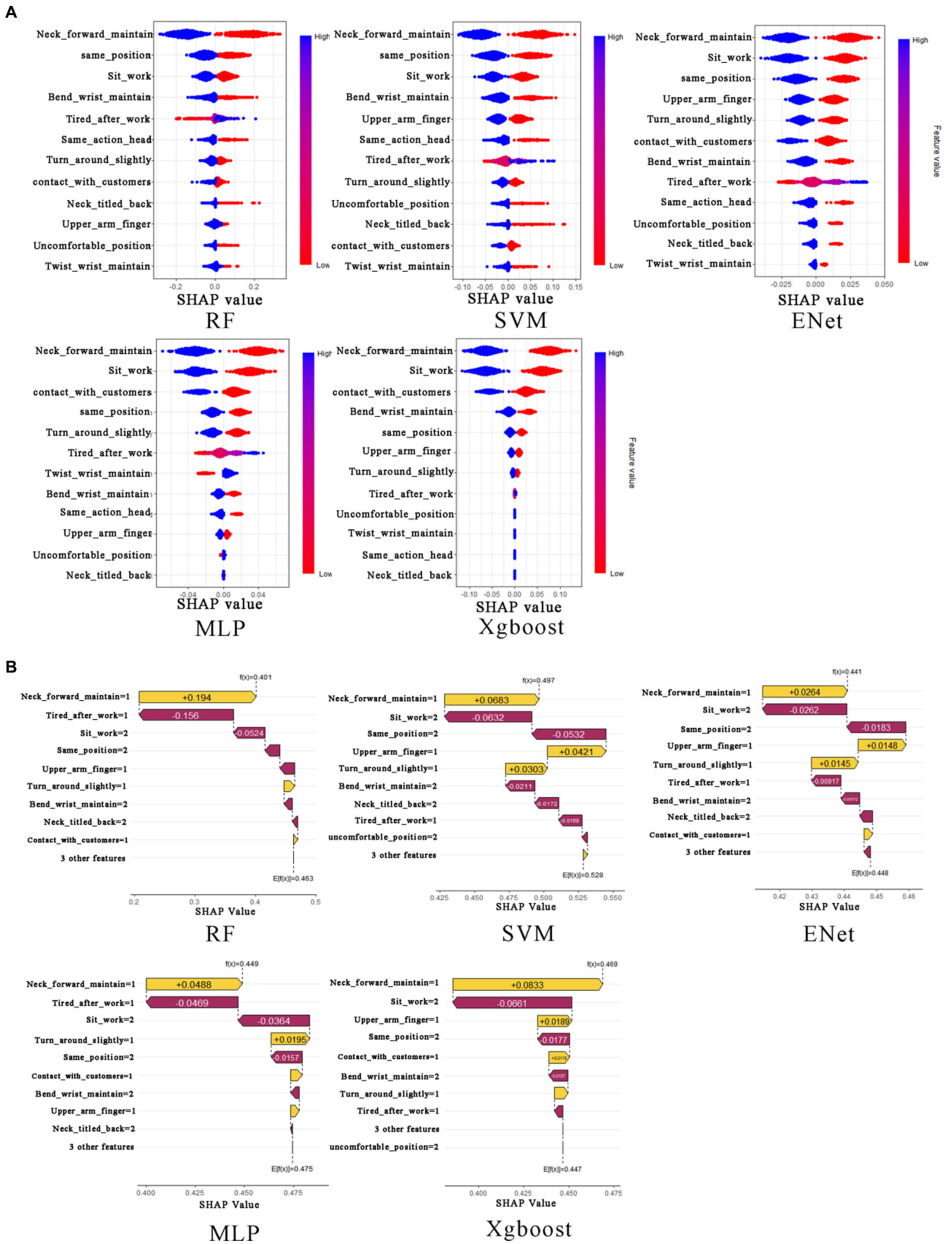
The beeswarm plot and waterfall plot for neck musculoskeletal disorders. In **(A)**, from the first row to the second row, and from left to right, the subplots represent the neck musculoskeletal disorders beeswarm plots for RF, SVM, Xgboost, Enet, and MLP, respectively. In **(B)**, from the first row to the second row, and from left to right, the subplots represent the neck musculoskeletal disorders waterfall plots for RF, SVM, Xgboost, Enet, and MLP, respectively.

**Figure 5:**
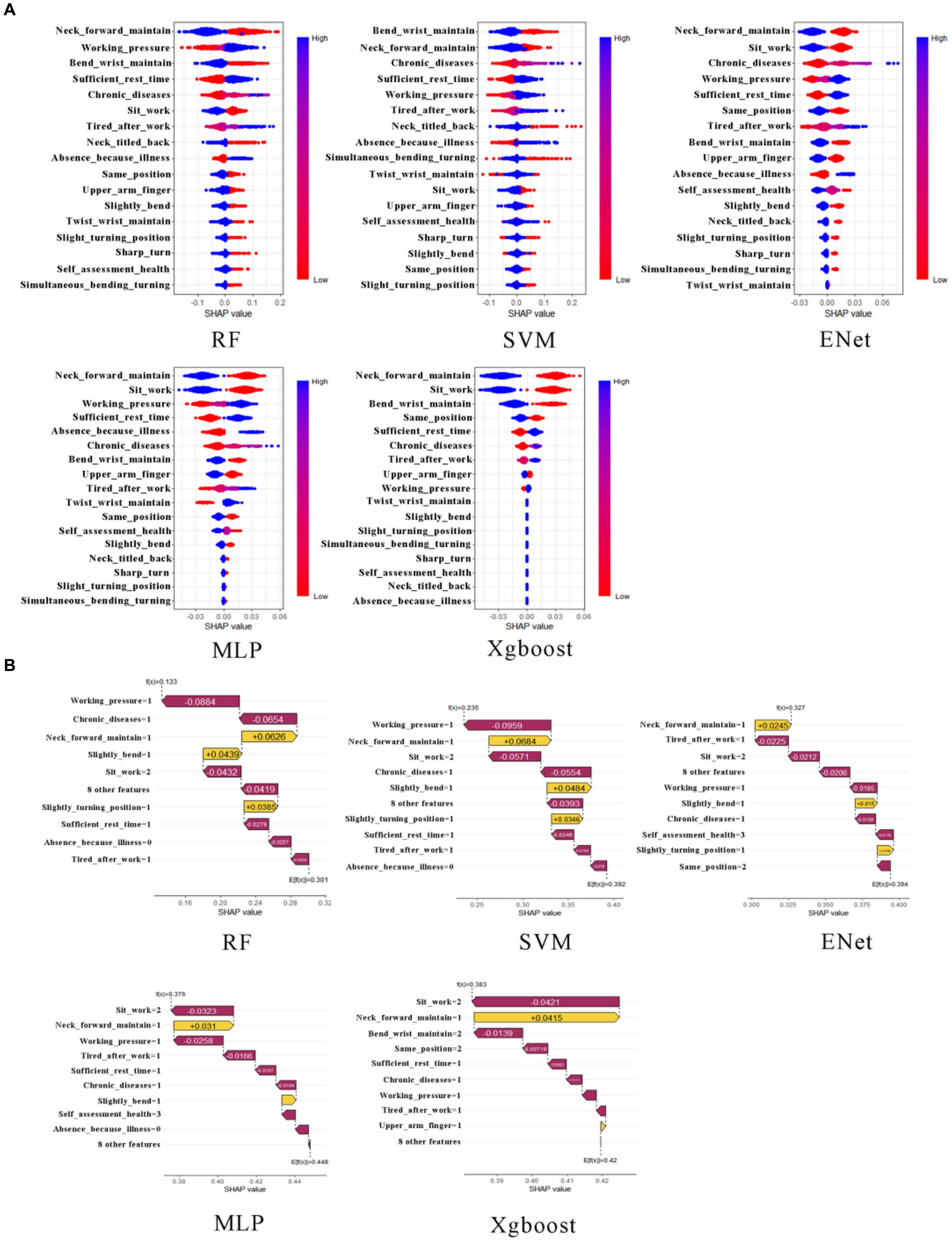
The beeswarm plot and waterfall plot for shoulder musculoskeletal disorders. In **(A)**, from the first row to the second row, and from left to right, the subplots represent the shoulder musculoskeletal disorders beeswarm plots for RF, SVM, Xgboost, Enet, and MLP, respectively. In **(B)**, from the first row to the second row, and from left to right, the subplots represent the shoulder musculoskeletal disorders waterfall plots for RF, SVM, Xgboost, Enet, and MLP, respectively.

We employ the SHapley Additive exPlanations (SHAP) framework as our chosen method for model interpretability. In this context, we utilize the R programming language and leverage both the “fastshap” and “shapviz” packages (10, 11). These tools allow us to construct beeswarm plots and waterfall plots, respectively. The bee swarm plots show the distribution of the SHAP values for each feature across all the data points, and the waterfall plots are individualized explanations of a single prediction, showing the contribution of each feature to the final prediction (see Figures 4, 5).

The Shapley value represents the average marginal contribution of a variable across all conceivable coalitions. For each individual, the SHAP value associated with each variable reflects its contribution to the individual’s risk of musculoskeletal disorders in the neck and shoulder. The determination of an individual’s susceptibility to neck and shoulder musculoskeletal disorders is achieved by summing the contributions of these variables relative to the baseline value (which corresponds to the average predicted age across the dataset).

#### 2.4.4 Partial dependency computation

**Figure 6:**
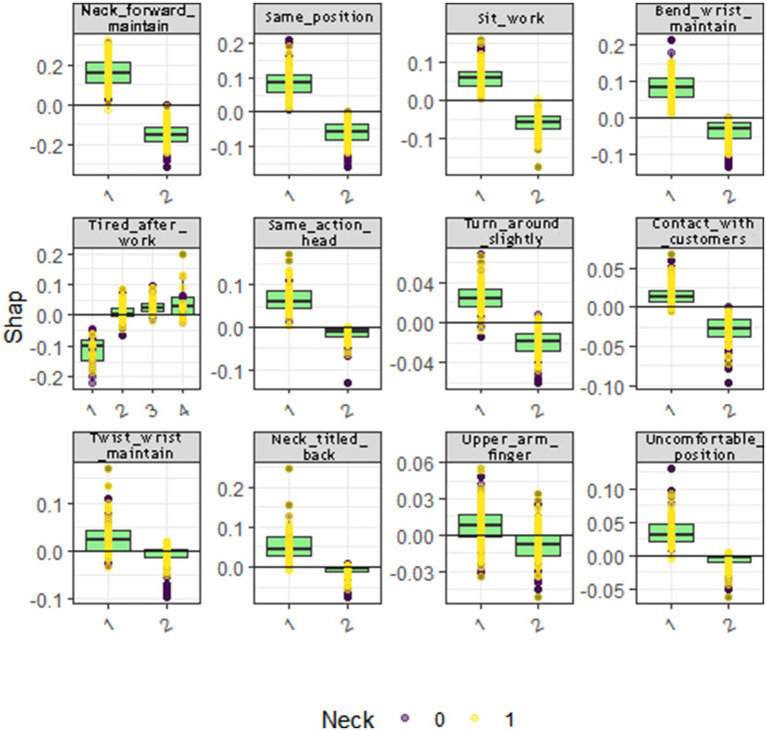
Bias plot of important factors for neck musculoskeletal disorders.

**Figure 7:**
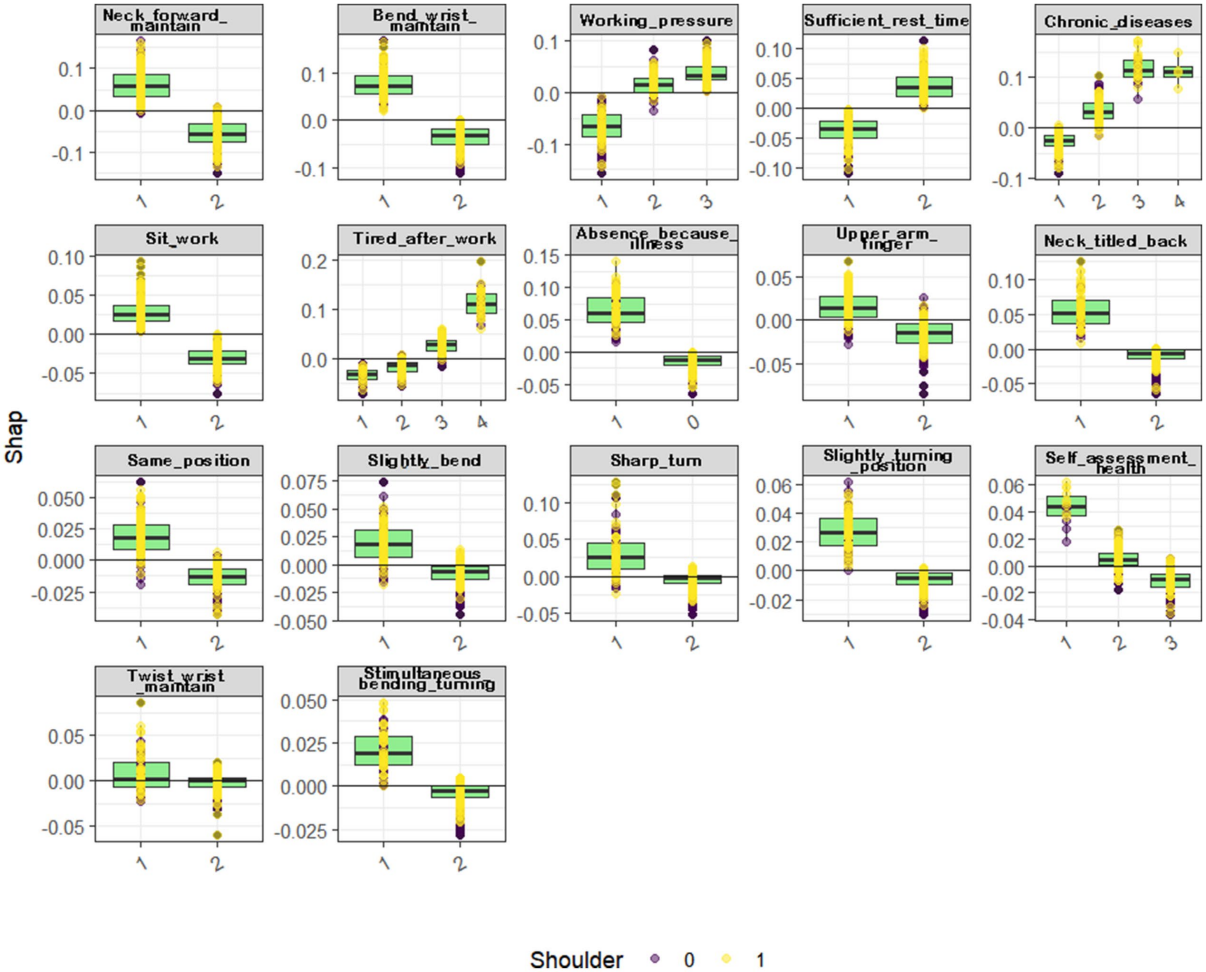
Bias plot of important factors for shoulder musculoskeletal disorders.

The computation and graphical representation of partial dependency values for each variable are showcased in Figures 6, 7, offering illustrative examples.

## 3 Results

### 3.1 Demographic data

The surveyed medical personnel consisted of 403 females and 214 males (see Table 2). Among them, 419 were married, 173 were unmarried, and 25 had unknown marital status. Regarding age distribution, 244 medical personnel were between 25 and 34 years old, 194 were between 35 and 44 years old, and 102 were between 45 and 54 years old. In terms of educational background, 42 respondents had education levels below a university degree, 527 had completed college or undergraduate studies, and 48 had completed postgraduate studies or above. Regarding work experience, 215 medical personnel had been in the profession for 15 years or more. Self-assessment of health status revealed that 379 individuals rated their health as average, while 216 rated it as good. As for monthly income, 201 medical personnel earned between 3,000 and 4,999 yuan, and 190 earned between 5,000 and 6,999 yuan. In terms of the size of their employing institutions, 376 medical personnel worked in units with 300–999 employees. Night shifts were part of the work schedule for 280 medical personnel. Additionally, 214 individuals had a weekly working time of 40 h or less, and 402 had no more than two types of chronic diseases.

**Table 2:** Basic demographic characteristics of survey participants.

	Total (%)	Neck musculoskeletal disorders		*X* ^2^	*p*	Shoulder musculoskeletal disorders		*X* ^2^	*p*
		Yes (%)	No (%)			Yes	No		
Gender				6.572	0.010			6.999	0.008
Male	214 (34.68)	81 (29.24)	133 (39.12)			69 (28.40)	145 (38.77)		
Female	403 (65.32)	196 (70.76)	207 (60.88)			174 (71.60)	229 (61.23)		
Marital status				1.780	0.411			3.901	0.142
Married	419 (67.91)	193 (69.68)	226 (66.47)			173 (71.19)	246 (65.78)		
Unmarried	173 (28.04)	71 (25.63)	102 (30.00)			58 (23.87)	115 (30.75)		
Other	25 (4.05)	13 (4.69)	12 (3.53)			12 (4.94)	13 (3.48)		
Age				4.321	0.364			5.284	0.259
<25 years	57 (9.24)	19 (6.86)	38 (11.18)			14 (5.76)	41 (10.96)		
25–34 years	244 (39.55)	107 (38.63)	137 (40.29)			93 (38.27)	151 (40.37)		
35–44 years	194 (31.44)	93 (33.57)	101 (29.71)			79 (32.51)	115 (30.75)		
45–54 years	102 (16.53)	49 (17.69)	53 (15.59)			47 (19.34)	55 (14.71)		
≥55 years	20 (3.24)	9 (3.25)	11 (3.24)			8 (3.29)	12 (3.21)		
Education				0.510	0.775			0.456	0.796
Below University	42 (6.81)	17 (6.14)	25 (7.35)			17 (7.00)	25 (6.68)		
College/undergraduate	527 (85.41)	237 (85.56)	290 (85.29)			205 (84.36)	322 (86.10)		
Postgraduate	48 (7.78)	23 (8.30)	25 (7.35)			21 (8.64)	27 (7.22)		
Work experience				5.626	0.131			10.487	0.015
<5 years	154 (24.96)	59 (21.30)	95 (27.94)			48 (19.75)	106 (28.34)		
5–9 years	126 (20.42)	53 (19.13)	73 (21.47)			43 (17.70)	83 (22.19)		
10–14 years	122 (19.77)	62 (22.38)	60 (17.65)			54 (22.22)	68 (18.18)		
≥15 years	215 (34.85)	103 (37.18)	112 (32.94)			98 (40.33)	117 (31.28)		
Self-rated health				12.849	0.002			8.726	0.013
Poor	22 (3.57)	12 (4.33)	10 (2.94)			10 (4.12)	12 (3.21)		
Fair	379 (61.43)	189 (68.23)	190 (55.88)			165 (67.90)	214 (57.22)		
Good	216 (35.01)	76 (27.44)	140 (41.18)			68 (27.98)	148 (39.57)		
Monthly income				5.602	0.231			7.404	0.116
<3,000 yuan	58 (9.40)	20 (7.22)	38 (11.18)			17 (7.00)	41 (10.96)		
3,000–4,999 yuan	201 (32.58)	84 (30.32)	117 (34.41)			71 (29.22)	130 (34.76)		
5,000–6,999 yuan	190 (30.79)	89 (32.13)	101 (29.71)			81 (33.33)	109 (29.14)		
7,000–8,999 yuan	83 (13.45)	43 (15.52)	40 (11.76)			40 (16.46)	43 (11.50)		
≥9,000 yuan	85 (13.78)	41 (14.80)	44 (12.94)			31 (12.76)	51 (13.64)		
Institution size				1.287	0.732			1.826	0.609
<20 employees	25 (4.05)	9 (3.25)	16 (4.71)			8 (3.29)	17 (4.55)		
20–299 employees	98 (15.88)	42 (15.16)	56 (16.47)			34 (13.99)	64 (17.11)		
300–999 employees	376 (60.94)	174 (62.82)	202 (59.41)			153 (62.96)	223 (59.63)		
≥1,000 employees	118 (19.12)	52 (18.77)	66 (19.41)			48 (19.75)	70 (18.72)		
Shift work				2.332	0.312			4.418	0.110
Yes, with night	280 (45.38)	118 (42.60)	162 (47.65)			100 (41.15)	180 (48.13)		
Yes, without night	159 (25.77)	79 (28.52)	80 (23.53)			73 (30.04)	86 (22.99)		
No	178 (28.85)	80 (28.88)	98 (28.82)			70 (28.81)	108 (28.88)		
Weekly work hours				15.731	0.001			15.204	0.002
≤40 h	214 (34.68)	81 (29.24)	133 (39.12)			65 (26.75)	149 (39.84)		
41–44 h	155 (25.12)	64 (23.10)	91 (26.76)			60 (24.69)	95 (25.40)		
45–48 h	110 (17.83)	66 (23.83)	44 (12.94)			56 (23.05)	54 (14.44)		
49–54 h	138 (22.37)	66 (23.83)	72 (21.18)			62 (25.51)	76 (20.32)		
Chronic diseases				20.162	0.000			23.053	0.000
0	402 (65.15)	156 (56.32)	246 (72.35)			136 (55.97)	166 (44.39)		
1	176 (28.53)	95 (34.30)	81 (23.82)			80 (32.92)	96 (25.67)		
2	33 (5.35)	21 (7.58)	12 (3.53)			22 (9.05)	11 (2.94)		
≥3	6 (0.97)	5 (1.81)	1 (0.29)			5 (2.06)	1 (0.27)		

### 3.2 Model performance comparison

**Figure 8:**
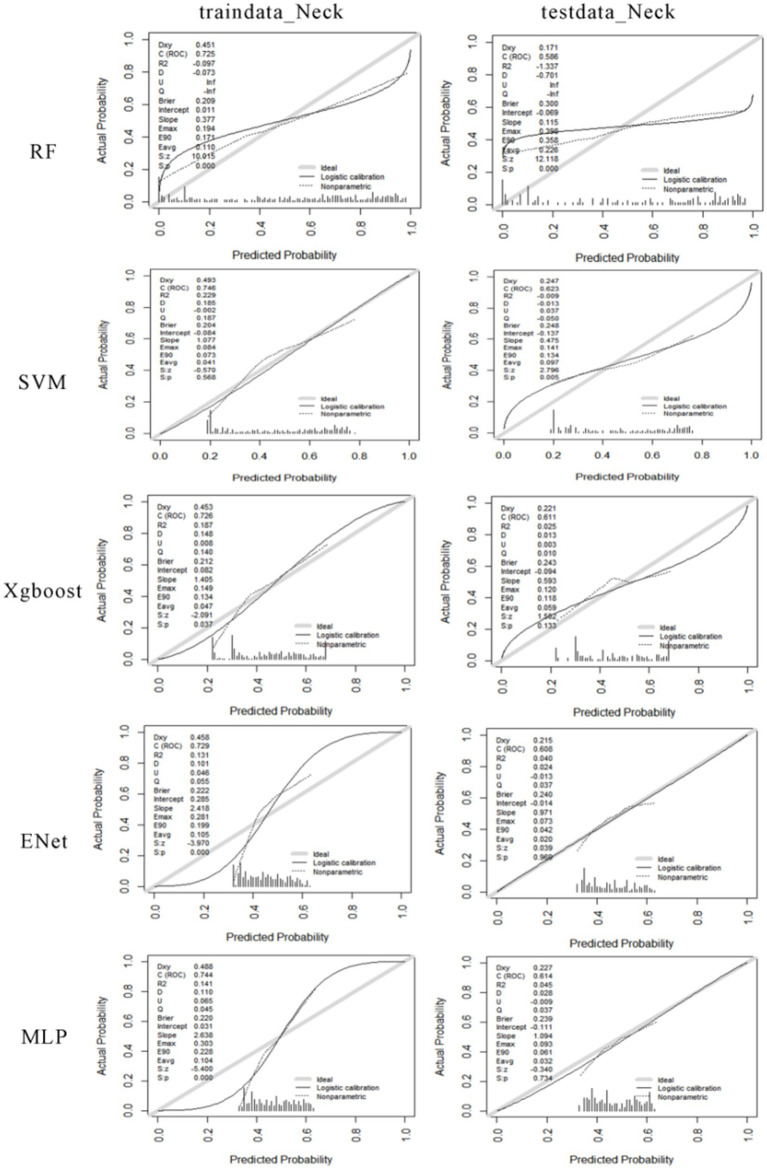
The calibration curves for machine learning models predicting the risk of neck musculoskeletal disorders. The first column shows the calibration curves for the training data, and the second column shows the calibration curves for the testing data. From the first row to the fifth row, the calibration curves for RF, SVM, Xgboost, Enet, and MLP are displayed for both the training and testing data.

The calibration curve of the model illustrates the degree of calibration in predicting probabilities on both the training and testing datasets. An ideal calibration model would exhibit a curve closely aligned with the diagonal line running from the lower-left corner to the upper-right corner. As the calibration curve approaches this diagonal line, the model’s probability predictions become more accurate. Performance varies among risk prediction models for different musculoskeletal disorders affecting the neck. The random forest model shows a relatively significant deviation from the ideal diagonal line on the training set, suggesting potential overfitting to the training data. On the other hand, the support vector machine exhibits a curve on the training set that is closer to the ideal diagonal line, indicating more accurate probability predictions. XGBoost demonstrates good calibration on the training data but appears to overestimate probabilities on the testing data. The calibration curve on the testing set for the elastic net model suggests a degree of miscalibration in predicting neck diseases. Although the MLP model exhibits strong calibration on the training data, its calibration performance on the testing data is comparatively subpar (see Figure 8).

**Figure 9:**
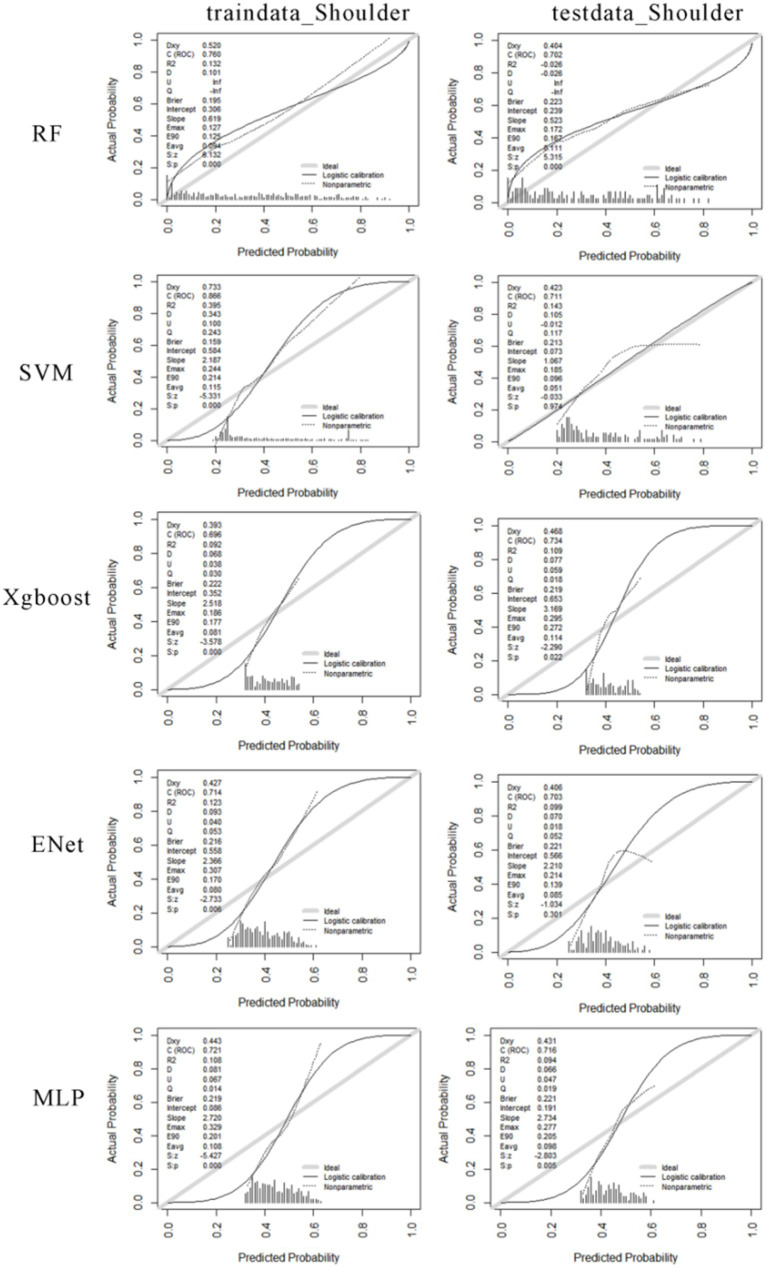
The calibration curves for machine learning models predicting the risk of shoulder musculoskeletal disorders. The first column shows the calibration curves for the training data, and the second column shows the calibration curves for the testing data. From the first row to the fifth row, the calibration curves for RF, SVM, Xgboost, Enet, and MLP are displayed for both the training and testing data.

The performance of various risk prediction models for different shoulder musculoskeletal disorders varies. The RF model exhibits a certain degree of deviation from the ideal diagonal line in both the training and testing calibration curves. This suggests some inconsistency between the model’s predicted probabilities and the actual occurrence frequencies. The SVM model displays a calibration curve close to the ideal state on the training set, indicating relatively accurate probability predictions during the training phase. However, the calibration curve on the testing set deviates slightly, indicating that the model’s predicted probabilities may be too high or too low when dealing with new data. The XGBoost model demonstrates good probability calibration on the training set, with a calibration curve that closely aligns with the ideal diagonal line. On the testing set, although there is a slight deviation in the curve, the overall performance remains relatively robust. The ENet model exhibits low predicted probabilities on both datasets, as evidenced in the histograms, where a significant portion of predicted probabilities clusters in the lower probability value range. Regarding the MLP model, the calibration curve on the training set indicates strong probability calibration. However, on the testing set, the curve deviates slightly from the ideal diagonal line, suggesting the possibility of mild overfitting. The histograms reveal a more even distribution of predicted probabilities on the training set but a somewhat more concentrated distribution on the testing set (see Figure 9).

Among the risk prediction model for neck musculoskeletal disorders, the SVM model achieves the lowest average MAE of 0.9165, indicating the smallest average prediction error. Following closely are the MLP and RF models, with average MAE values of 0.9850 and 0.9855, respectively. The Xgboost model has a slightly higher average MAE of 0.9950. The ENet model exhibits the highest average MAE at 0.9990.

Similarly, the SVM model attains the lowest average RMSE of 1.0385, signifying its superior performance when considering penalties for larger errors. The MLP model follows with an average RMSE of 1.0940, ranking second. ENet, Xgboost, and RF models display similar RMSE values of 1.1010, 1.1035, and 1.1045, respectively.

Among these models, the SVM model excels in both MAE and RMSE, indicating its relatively high predictive accuracy, especially in handling larger prediction errors. The MLP model performs well in RMSE but slightly lags behind the SVM in MAE. ENet, XGBoost, and RF models exhibit comparable performance in both metrics but fall slightly short of SVM and MLP.

In the risk prediction model for shoulder musculoskeletal disorders, The MLP (Multilayer Perceptron) model shows the best performance on both the training set (MAE = 0.946) and the testing set (MAE = 0.954), indicating its predictions are closest to the actual values on average. The XGBoost model follows closely with MAE = 0.974 on the training set and MAE = 0.982 on the testing set, suggesting slightly less accurate predictions than MLP but still outperforming other models. The SVM and ENet models have identical MAE on the training set (MAE = 1.001) and very similar performance on the testing set (SVM MAE = 1.009, ENet MAE = 1.007), which are moderate compared to MLP and XGBoost. The RF (Random Forest) model exhibits the highest MAE, particularly on the testing set (MAE = 1.111), which implies less accurate predictions on average compared to the other models.

**Figure 10:**
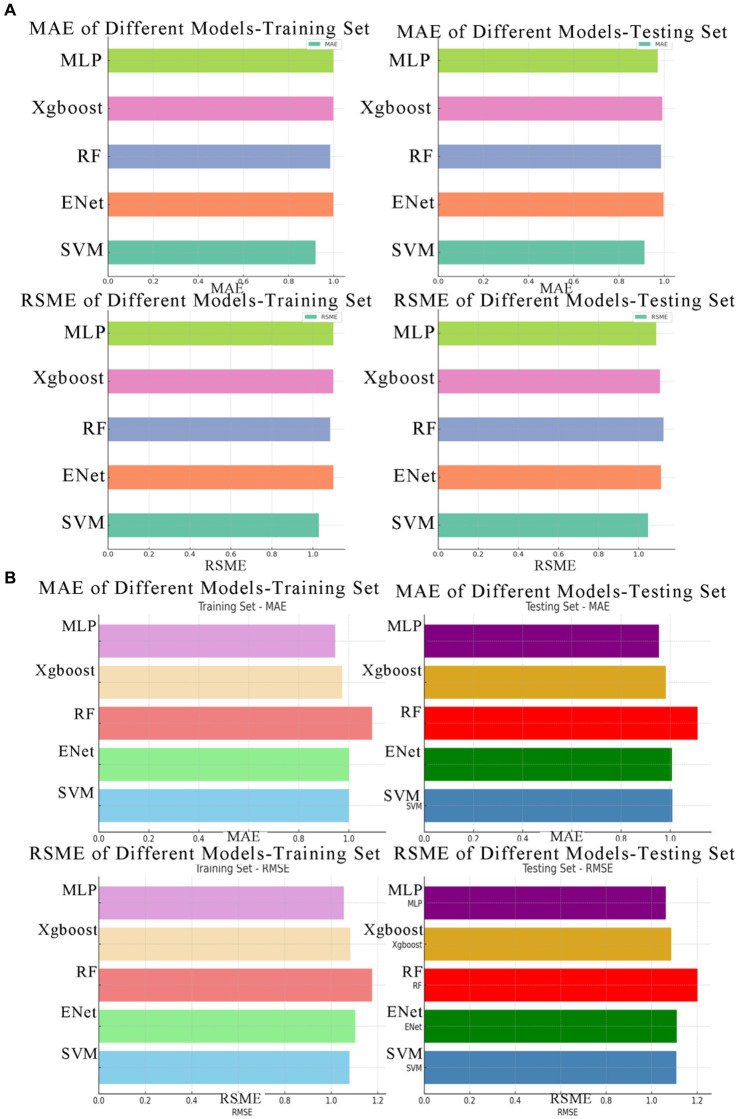
The MAE and RMSE values for various machine learning models. **(A)** Depicts the MAE and RMSE values for neck musculoskeletal disorders, while **(B)** illustrates the MAE and RMSE values for shoulder musculoskeletal disorders.

The MLP model stands out as the most consistent and accurate model for predicting shoulder musculoskeletal disorders according to both MAE and RMSE metrics. XGBoost also performs well and could be considered a good alternative, especially if computational efficiency is a concern, as gradient boosting can be more computationally intensive than neural networks depending on the implementation and dataset size. The SVM and ENet models show moderate performance, while the RF model might require further parameter tuning or feature engineering to improve its prediction accuracy (see Figure 10).

When evaluating various machine learning models for predicting neck musculoskeletal disorders, we observed that conventional logistic regression model performs relatively average. The Random Forest model exhibited relatively high overall performance on the training set (accuracy = 0.703, sensitivity = 0.749, specificity = 0.667, AUC = 0.772). However, on the testing set, the SVM model outperformed with an accuracy of 0.574 and an AUC of 0.623. This suggests that while the Random Forest model demonstrates strong learning capabilities during the training phase, the SVM model exhibits more stable performance when generalizing to unseen data (see Table 3).

**Table 3:** Comparison of model performance for neck musculoskeletal disorder prediction.

Neck musculoskeletal disorder prediction model	Accuracy	Sensitivity	Specificity	AUC
Logistic regression	0.668	0.756	0.570	0.718
SVM—Training set	0.684	0.865	0.537	0.746
SVM—Testing set	0.574	0.700	0.471	0.623
ENet—Training set	0.684	0.729	0.647	0.732
ENet—Testing set	0.587	0.629	0.553	0.616
RF—Training set	0.703	0.749	0.667	0.772
RF—Testing set	0.606	0.600	0.612	0.630
Xgboost—Training set	0.647	0.855	0.478	0.729
Xgboost—Testing set	0.568	0.786	0.388	0.610
MLP—Training set	0.673	0.691	0.659	0.741
MLP—Testing set	0.587	0.571	0.600	0.619

For the prediction of shoulder musculoskeletal disorders, the conventional logistic regression model performs relatively average. The SVM model demonstrates the best performance on the training set (accuracy = 0.781, sensitivity = 0.802, specificity = 0.768, AUC = 0.866). On the testing set, the MLP model achieves the highest accuracy (0.690), while the Xgboost model has the highest AUC value (0.734). This suggests that the SVM model exhibits strong fitting capabilities to the data during the training phase, but on the testing set, the MLP and Xgboost models provide better generalization. Particularly, the MLP model exhibits higher specificity (0.713) on the testing set, indicating its good performance in reducing false positives (see Table 4).

**Table 4:** Comparison of model performance for shoulder musculoskeletal disorder prediction.

Shoulder musculoskeletal disorder prediction model	Accuracy	Sensitivity	Specificity	AUC
Logistic regression	0.684	0.599	0.778	0.737
SVM—Training set	0.781	0.802	0.768	0.866
SVM—Testing set	0.665	0.705	0.638	0.712
ENet—Training set	0.677	0.560	0.754	0.714
ENet—Testing set	0.677	0.492	0.798	0.703
RF—Training set	0.690	0.797	0.621	0.775
RF—Testing set	0.645	0.721	0.596	0.719
Xgboost—Training set	0.639	0.720	0.586	0.696
Xgboost—Testing set	0.658	0.656	0.660	0.734
MLP—Training set	0.667	0.665	0.668	0.721
MLP—Testing set	0.690	0.656	0.713	0.716

### 3.3 Interpretability of machine learning models for the risk of musculoskeletal disorders

To quantitatively delineate the contribution of each variable in predicting the risk of musculoskeletal disorders of the neck, our investigation primarily focuses on the application of the Shapley Additive Explanations (SHAP) framework within the Random Forest (RF) and Support Vector Machine (SVM) models. The RF model elucidates the top six determinants impacting the susceptibility of Healthcare Professionals to musculoskeletal disorders: prolonged forward neck posture, wrist flexion or maintenance of this position for extended periods, physical exhaustion post-work, prolonged neck twisting posture, static posture maintenance, and prolonged sedentary work. The SVM model reveals a similar hierarchy of influential factors, albeit with slight variations in their order. The results of conventional logistic regression (LR) are shown in Table 5, but since the performance of LR is inferior to that of random forest (RF) and support vector machine (SVM), it is not discussed in detail. Notably, the sustained forward tilt of the wrist significantly augments the risk of neck-related musculoskeletal disorders. Conversely, prolonged sitting and maintaining a uniform posture while working exhibit a negative correlation with the risk of developing these disorders (refer to Figure 4).

**Table 5:** Neck musculoskeletal disorder binary logistic regression results.

Item	*B*	Sig	OR	95%CI interval
Do you feel physically tired after work? (ref: very tired)				
Not tired	−1.225	0.007	0.294	0.121 ~ 0.711
A little	−0.328	0.377	0.720	0.348 ~ 1.492
Tired	−0.078	0.842	0.925	0.429 ~ 1.996
Does your job frequently involve interaction with patients or the public?	0.441	0.026	1.554	1.054 ~ 2.291
Do you often slightly turn your body during work?	−0.164	0.540	0.849	0.502 ~ 1.436
Do you frequently lean your neck forward or maintain this posture for extended periods during work?	0.812	<0.001	2.253	1.457 ~ 3.483
Do you frequently lean your neck backward or maintain this posture for extended periods during work?	0.151	0.620	1.163	0.640 ~ 2.111
Do you frequently bend your wrists or maintain this posture for extended periods during work?	0.243	0.356	1.275	0.761 ~ 2.138
Do you frequently twist your wrists and maintain this posture for extended periods during work?	−0.423	0.180	0.655	0.353 ~ 1.216
Do you frequently repeat the same movements with your upper arms and fingers multiple times per minute during work?	0.027	0.904	1.028	0.657 ~ 1.607
Do you frequently repeat the same movements with your head multiple times per minute during work?	0.126	0.635	1.135	0.673 ~ 1.912
Do you often work in uncomfortable postures?	0.116	0.670	1.123	0.659 ~ 1.912
Do you spend long periods sitting during work?	0.559	0.004	1.749	1.191 ~ 2.569
Do you maintain the same posture for extended periods during work?	0.179	0.425	1.196	0.770 ~ 1.857

To quantitatively exhibit the contribution of each variable in the prediction of shoulder musculoskeletal disorder risks, we primarily examine the outcomes of the Shapley Additive Explanations (SHAP) tree framework on the Multilayer Perceptron (MLP) and Support Vector Machine (SVM) models. The MLP model identifies the six principal factors affecting the risk among Healthcare Professionals: prolonged forward neck posture, prolonged sedentary work, work-related stress levels, number of chronic diseases, physical exhaustion post-work, and absence due to illness. Conversely, the SVM model highlights the top six influential factors as prolonged neck twisting posture, number of chronic diseases, sustained bending posture, absence due to illness, physical exhaustion post-work, and wrist flexion or maintenance of this position for extended periods. The results of conventional logistic regression (LR) are shown in Table 6, but since the performance of LR is inferior to that of multilayer perceptron (MLP) and support vector machine (SVM), it is not discussed in detail. Notably, low levels of work stress and not sitting for prolonged durations have a negative impact on the risk of lumbar musculoskeletal disorders. In contrast, maintaining a prolonged forward neck posture significantly increases the risk of shoulder musculoskeletal disorders (refer to Figure 5).

**Table 6:** Shoulder musculoskeletal disorder binary logistic regression results.

Item	*B*	Sig	OR	95%CI interval
Do you feel physically tired after work? (ref: very tired)				
Not tired	−0.934	0.058	0.393	0.150 ~ 1.033
A little	−0.480	0.216	0.619	0.289 ~ 1.324
Tired	−0.410	0.309	0.664	0.301 ~ 1.462
Do you frequently lean your neck forward or maintain this posture for extended periods during work?	0.744	0.002	2.105	1.316 ~ 3.367
Do you frequently lean your neck backward or maintain this posture for extended periods during work?	−0.060	0.845	0.942	0.516 ~ 1.720
Do you frequently bend your wrists or maintain this posture for extended periods during work?	0.303	0.261	1.354	0.798 ~ 2.297
Do you frequently twist your wrists and maintain this posture for extended periods during work?	−0.602	0.064	0.547	0.290 ~ 1.035
Do you frequently repeat the same movements with your upper arms and fingers multiple times per minute during work?	0.245	0.288	1.278	0.813 ~ 2.011
Do you spend long periods sitting during work?	0.630	0.003	1.877	1.248 ~ 2.824
Do you maintain the same posture for extended periods during work?	0.013	0.956	1.013	0.643 ~ 1.595
Self-assessment of your health status (ref: very good)				
Poor	−0.172	0.748	0.842	0.295 ~ 2.404
Average	0.080	0.706	1.083	0.717 ~ 1.636
Have you taken sick leave in the past year due to illness?	−0.165	0.482	0.848	0.536 ~ 1.343
Work-related stress (ref: high)				
Low	−0.467	0.047	0.627	0.395 ~ 0.994
Average	−0.056	0.805	0.945	0.605 ~ 1.477
Types of chronic diseases (ref: 3 or more)				
0	−2.060	0.074	0.127	0.013 ~ 1.220
1	−1.788	0.123	0.167	0.017 ~ 1.619
2	−0.987	0.415	0.373	0.035 ~ 4.008
Do you feel your rest periods are sufficient?	−0.359	0.070	0.699	0.474 ~ 1.030
Do you frequently make large turns of your body during work?	0.230	0.435	1.259	0.706 ~ 2.243
Do you frequently maintain a slightly bent posture for extended periods during work?	−0.304	0.307	0.738	0.412 ~ 1.322
Do you frequently maintain a slightly turned posture for extended periods during work?	0.016	0.964	1.016	0.501 ~ 2.061
Do you frequently maintain a posture that involves both bending and turning for extended periods during work?	0.241	0.539	1.273	0.590 ~ 2.744

Healthcare professionals who maintain a prolonged forward neck posture face a higher risk of developing neck musculoskeletal disorders. Similarly, those with extended periods of wrist flexion are more likely to suffer from these disorders. Medical staff experiencing varying degrees of tiredness post-work—from slightly tired to extremely exhausted—are more susceptible to neck musculoskeletal disorders. Additionally, a long-term neck twisting posture and prolonged periods of sitting while working significantly increase the likelihood of these conditions (refer to Figure 6).

Results from the SVM and MLP models indicate that healthcare professionals who frequently maintain a forward neck posture are at a greater risk of shoulder musculoskeletal disorders. Similarly, prolonged sitting while working elevates the risk of these disorders. Moderate to high levels of work-related stress are more likely to lead to shoulder musculoskeletal disorders in medical staff. Those with one or more types of chronic diseases face a heightened risk of developing these conditions. Experiencing tiredness or extreme fatigue after work increases the likelihood of these disorders, as does a history of absenteeism due to illness. Moreover, maintaining a prolonged neck twisting posture, sustaining a significant bending posture for extended periods, and long-term wrist flexion are all associated with an increased risk of shoulder musculoskeletal disorders (see Figure 7).

## 4 Discussion

Different models exhibit varying performances in assessing the risk of shoulder and neck musculoskeletal disorders, each with unique strengths and limitations. For instance, while the Random Forest excel in training datasets for predicting neck musculoskeletal disorder risks, the SVM demonstrate superior generalization abilities on test datasets. These findings emphasize the importance of considering performance metrics when selecting models for specific medical prediction tasks, especially in clinical applications where a model’s generalizability and its ability to reduce misdiagnosis (through high specificity) are crucial. Future research could explore these models’ performances on larger and more diverse datasets and refine their parameter settings, offering deeper insights for effective clinical prediction of musculoskeletal diseases.

Many studies using machine learning models lack interpretability (12–14), making it challenging to verify their reliability. Interpretability supports the acceptability of evidence and facilitates data-driven, personalized healthcare management. To achieve this, we have developed interpretable models for predicting the risk of shoulder and neck musculoskeletal disorders, utilizing SHAP algorithms for individual-level local explanations. Past studies have emphasized occupational factors as the main contributors to WMSDs, where muscle activity and movement during occupational tasks can lead to their occurrence. This finding is consistent with the results of this study, where the most critical influencing factors for neck and shoulder musculoskeletal disorders were occupational factors (15–20).

Currently, the predominant method for musculoskeletal disorder screening in China utilizes the Chinese version of the “Musculoskeletal Disorder Questionnaire” provided by the Occupational Health and Poison Control Institute of the Chinese Center for Disease Control and Prevention. This comprehensive questionnaire, consisting of 133 items requiring 5–10 min to complete, was modified by Dong et al. into the Chinese Musculoskeletal Questionnaire (CMQ) (21), which includes five major categories and 48 items. However, it remains time-consuming for occupational screening. Our study employs the Boruta algorithm for feature selection, reducing neck musculoskeletal disorder screening to just 12 key items and shoulder disorder screening to 17, Enabling a simplified screening process to identify individuals at higher risk of musculoskeletal disorders. By inputting demographic data into an electronic system, the musculoskeletal disorder prediction model can assess the risk of these conditions in healthcare professionals, thereby significantly reducing the workload for screening.

It is noteworthy that the forward posture of the neck in healthcare professionals significantly contributes to the risk of musculoskeletal disorders in both the neck and shoulder regions. Providing ergonomic chairs are recommended. Zhang et al. found that factors influencing sonographer’s physicians’ musculoskeletal disorders include work duration, consistent with the results of this study, where work duration was the main influencing factor for shoulder musculoskeletal disorders among healthcare professionals (12). We recommend providing targeted ergonomics-oriented occupational health education for medical staff, replacing ergonomic chairs, encouraging correct working postures, and emphasizing the importance of rest after work to reduce the incidence of occupational musculoskeletal disorders. Personalized musculoskeletal disorder risk management advice should be provided to healthcare professionals across different departments, considering both occupational factors and individual health profiles. In addition to professional factors, this study also discovered a correlation between the number of chronic diseases in medical personnel and the risk of shoulder musculoskeletal disorders, suggesting that future research should delve deeper into the clinical mechanisms linking work-related musculoskeletal disorders with chronic diseases.

This study also has certain limitations. The absence of physiological tests makes it difficult to eliminate factors causing musculoskeletal disorders unrelated to work. Another limitation is the lack of comparison of musculoskeletal disorder factors among medical staff from different departments. The risk prediction models are derived from cross-sectional data, where exposure and outcome are ascertained at the same time point, inherently limiting the predictions. Additionally, the sample size of this study is relatively small. Future studies should establish large cohorts of healthcare workers with WMSDs to better explore the causal relationships between variables. Furthermore, a comparative analysis of musculoskeletal disorder factors among medical staff from different departments should be conducted.

## 5 Conclusion

Five machine learning models were utilized to construct predictive models for the risk of neck and shoulder musculoskeletal disorders among healthcare professionals. These models are economically feasible and convenient for preliminary screening of work-related musculoskeletal disorders in healthcare workers. Additionally, this study offers a comprehensive interpretable machine learning framework, enabling a quantitative analysis of the impact of occupational factors on the risk of work-related musculoskeletal disorders. A web calculator can be applied to the early detection and prevention of neck and shoulder WMSDs in healthcare workers.

## Data Availability

The raw data supporting the conclusions of this article will be made available by the authors, without undue reservation.
